# District decision-making for health in low-income settings: a systematic literature review

**DOI:** 10.1093/heapol/czv124

**Published:** 2016-09-01

**Authors:** Deepthi Wickremasinghe, Iram Ejaz Hashmi, Joanna Schellenberg, Bilal Iqbal Avan

**Affiliations:** ^1^IDEAS Project, London School of Hygiene & Tropical Medicine, UK

**Keywords:** Decision-making, evidence-based policy, low-income, health systems, decentralization, health planning

## Abstract

Health management information systems (HMIS) produce large amounts of data about health service provision and population health, and provide opportunities for data-based decision-making in decentralized health systems. Yet the data are little-used locally. A well-defined approach to district-level decision-making using health data would help better meet the needs of the local population. In this second of four papers on district decision-making for health in low-income settings, our aim was to explore ways in which district administrators and health managers in low- and lower-middle-income countries use health data to make decisions, to describe the decision-making tools they used and identify challenges encountered when using these tools. A systematic literature review, following PRISMA guidelines, was undertaken. Experts were consulted about key sources of information. A search strategy was developed for 14 online databases of peer reviewed and grey literature. The resources were screened independently by two reviewers using pre-defined inclusion criteria. The 14 papers included were assessed for the quality of reported evidence and a descriptive evidence synthesis of the review findings was undertaken. We found 12 examples of tools to assist district-level decision-making, all of which included two key stages—identification of priorities, and development of an action plan to address them. Of those tools with more steps, four included steps to review or monitor the action plan agreed, suggesting the use of HMIS data. In eight papers HMIS data were used for prioritization. Challenges to decision-making processes fell into three main categories: the availability and quality of health and health facility data; human dynamics and financial constraints. Our findings suggest that evidence is available about a limited range of processes that include the use of data for decision-making at district level. Standardization and pre-testing in diverse settings would increase the potential that these tools could be used more widely.

Key Messages

- Health management information systems produce large amounts of data, yet little data are used locally in health decision-making.
- This systematic literature review explored ways in which district administrators and health managers in low- and lower-middle-income countries used health data to make decisions, to describe the decision-making tools they used and identify challenges encountered when using these tools.
- Our findings suggest that evidence is available about a limited range of processes that include the use of data for decision-making at district level.

## Introduction

Health management information systems (HMIS) produce data about health service provision and population health status that are intended to be used for decision-making and planning at all levels of the health system, especially in the local area where they have been generated. Examples from research studies to encourage the use of local health data at community level include: a randomized field experiment in Uganda to encourage community monitoring of health services, in which the community used health data to hold their local health workers to account for performance, leading to greater utilization of health services and improved health outcomes (Björkman and Svensson 2009); and a participatory approach to community assessment and planning for maternal and child health programmes in Ethiopia, which resulted in health data and community priorities being used to decide health care activities (Bhattacharyya and Murray 2000). Yet in practice HMIS data are not being used enough at community or district level. One reason for this might be that there is no standardized process for their usage (Harrison and Nutley 2010; Qazi and Ali 2011), or alternatively, data may not be available, maybe incomplete or of poor quality (Braa *et al.* 2012; Nutley 2012).

When considering information use in organizations, Feldman and March (1981) identified wider impediments to using data rationally for decision-making, which might also be applied to the field of health administration. These are based on users’ perceptions that the data are inadequate or irrelevant, because the data gathers and users are two distinct groups; the data have been collected for a different purpose, e.g. for monitoring rather than decision-making; the data are subject to strategic misrepresentation; or that using data as a symbol of rational decision-making takes on more significance than the outcome of the process.

Yet the formal use of local data can help in setting district health priorities and planning, resource allocation and utilization, managing health workers and introducing new services or improving existing service delivery and quality to better meet the needs of the local population (Smith *et al.* 1989; Gill and Bailey 2010; Chitama *et al.* 2011). HMIS provide opportunities for data-based decision-making and are designed for use within decentralized health systems, which are amenable to decision-making at district level (Smith *et al.* 1989; Kimaro and Sahay 2007; Qazi *et al.* 2008; Talukder *et al.* 2008).

The extent to which local public health administrators—those working at district-level, or the equivalent—are able to make health decisions and undertake planning locally is important and is closely linked to strategies for health services administration, such as decentralization (Bossert *et al.* 2000; Nyamtema 2010). Without some degree of decentralization, local administrators are not able to make meaningful decisions that they can follow through to benefit the community (Bossert and Beauvais 2002).

Much has been written about health systems data collection and ways to improve data quality, e.g. (Braa *et al.* 2007; Abajebel *et al.* 2011; Chitama *et al.* 2011) but what is less well documented is how the data are used. This review explored local decision-making practices in low- and lower-middle-income countries and the types of evidence used to make those decisions. Decision-making in health systems administration is the process by which a group of people reach a collective understanding of a topic, which then helps to build consensus on a particular course of action to address a health service challenge, from two or more possible options. In a rational decision-making process, all the options available are given full and unbiased consideration; relevant data and information are assessed; expertise and experience—either from within the group or from an external source—are drawn upon; the expected result of following each option is assessed; and the option most likely to be successful is chosen (Stone 2012). Ideally, decision-making is based on a full assessment of all the available data that meet accepted quality criteria. This is widely used within the health sector as the standard way in which clinical decisions are made, yet within health systems, data do not always form the basis for managerial or administrative decisions (Pappaioanou *et al.* 2003; Walshe and Rundall 2001).

From the literature, we sought to identify well-defined ways that data are used locally, through structured processes, tools or guidelines that facilitate the various district level stakeholders to make decisions, and whether any of these processes had been standardized through pretesting and piloting. We consider structured decision-making processes to be those that contain predefined steps, include a consensus building process and incorporate the use of locally generated data.

This is the second paper in a series of four: the first is on the feasibility of establishing a data-informed platform for health to support district data for decision-making in India, Nigeria and Ethiopia (Avan *et al.* 2016); the third paper presents potential data sources using the World Health Organization’s health-system block framework, showing the huge potential of HMIS data at district level in India and Ethiopia (Bhattacharyya *et al.* 2016) and the final paper in the series presents prospects for engaging the private sector in health data sharing and collaborative decision-making at district level in India (Gautham *et al.* 2016).

The objective of this systematic literature review was to look at the ways in which local administrators and managers in the health system—at district level or the equivalent—in low- and lower-middle-income countries, use health data to make decisions. Our aims were to identify and describe the decision-making processes, tools or guidelines used to make decisions that led to changes in local health systems; and understand the key steps within these processes, any common steps in the different processes, and any challenges that affected their implementation.

## Methods

The PRISMA 2009 statement and checklist (Moher *et al.* 2009) were adopted, to ensure this systematic literature review followed a transparent, replicable and iterative process. The date, activities and outcome of each step of the process were recorded in a log of activities. The protocol was published online in the PROSPERO international database of prospectively registered systematic reviews in health and social care, at the Centre for Reviews and Dissemination, University of York, on 3 October 2013; registration number: CRD42013005306 (Center for Reviews Dissemination).

### Eligibility criteria

This review comprised qualitative papers and reports, including literature reviews and case studies that described formal decision-making processes used at district level, incorporating the use of health systems data; detailed the steps in an effective process; identified decision-making instruments available and evaluated the effectiveness of a decision-making process. The focus was local health systems administration decision-making, in low- or lower-middle-income countries as classified by the World Bank in 2012 (World Bank 2012). Studies about data collection issues were excluded from this review, as were those that focused on either the process, or the impact of decentralizing of health systems.

### Search strategy and information sources

The literature search focused on formal decision-making by local administrators, in low- and lower-middle-income countries, using evidence and information from health systems data (a primary source of which is usually HMIS). While initiated in the public sector, a decision-making process may also involve other stakeholders, including private sector and non-governmental organizations (NGOs) responsible for delivering health services at district level and below.

A comprehensive literature search strategy was developed, incorporating the different elements of the enquiry: decision-making, evidence, district, health systems data and the countries in the inclusion criteria. It included both free text and medical subject headings (MESH terms). It was tested, reviewed and refined; and searches were conducted in 11 electronic databases of peer-reviewed work: EconLit, EMBASE, Global Health, Health Systems Evidence, HMIC (Health Management Information Consortium), LILACS (Health Science Literature from and relevant to Latin America and the Caribbean), MEDLINE, PsychINFO, Scopus, Social Policy and Practice, and Web of Science. MESH terms, or the equivalent, were tailored to individual databases. The search strategy for Medline is available in Supplementary file 1. A search for grey literature was conducted, through semi-structured interviews with experts in this field, to identify possible sources and documents, and a search of grey literature databases: Popline, New York Academy of Medicine Grey Literature Report and Google Scholar.

### Selection of studies

**Figure 1.**
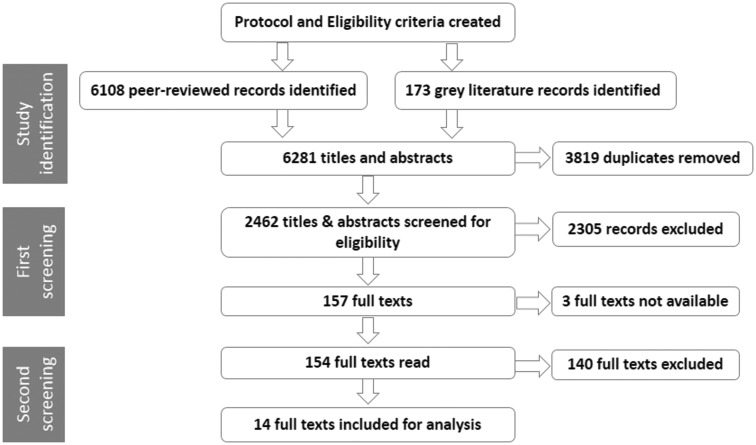
Flow diagram of the systematic review process.

Figure 1 is a flow diagram, showing the number of records at each stage of the systematic review process.

The literature searches were conducted, records were uploaded to EndNote X7 and duplicates were removed (D.W.).

The titles and abstracts were screened independently by two reviewers (initially D.W. and M.T., then D.W. and I.E.H.) to identify studies that met two criteria: that they related to a low- or lower-middle-income country and focused on the district level. Where either reviewer excluded a record, or was unsure, the reason was recorded in EndNote. They compared their decisions and reached consensus about which records should be included. Full texts were sought for records for definite inclusion and those marked unsure. All but three full texts were found, however each of these records had been marked as unsure during the initial screening. A standardized data extraction form was developed, (see Supplementary file 2) modified from the Health Care Provider Performance data extraction form produced by CDC Foundation (2015), and incorporating the three characteristics of a structured decision-making process—that it consists of a series of steps, includes a consensus process and uses locally generated data. For the second screening, the two reviewers divided the full texts and worked independently, reading them and completing the form for those relevant to the review and noting the reason for excluding studies and those they were unsure about whether to include in this review. Online database software was used to store the information. To ensure a consistent approach between both reviewers, each then read a random selection of 10 papers from the full texts they had not read initially and made an independent decision about whether or not they should be included. The two reviewers then discussed all the studies that either was unsure about including in the review, to check whether they met the eligibility criteria. They reached agreement about whether or not to include all but one paper, for which it was not very clear whether it met the criteria, and this was referred to a third reviewer (B.I.A.) for a final decision. Supplementary file 3 lists the 14 papers included for review, of which three are about the same study.

### Assessment of quality of evidence

To assess the quality of the reporting of evidence in the papers for review, a form was adapted from various existing quality assessment forms for qualitative research (National Institute for Health and Care Excellence 2007; Critical Appraisal Skills programme 2010; Tong *et al.* 2012). This consisted of 15 criteria covering features that might be expected in the abstract and introduction; study design; methodology; results and discussion sections of observational studies. Each question could be answered ‘yes’ or ‘no’. Adapting the Scottish Intercollegiate Guidelines Network (SIGN) levels (Scottish Intercollegiate Guidelines Network 2012), studies meeting over 75% of the quality criteria were considered to be of high quality ( +++), those with between 50% and 75% were considered to be of acceptable quality ( ++) and those with <50% were considered to be of low quality (+).

## Results

### Overview

Details of the quality assessment for the way each study was reported in the papers included in this review are given in Supplementary file 4; 10 papers were found to report a high quality of evidence, (Sandiford *et al.* 1994; Heinonen *et al.* 2000; Mubyazi *et al.* 2004; Chaulagai *et al.* 2005; Mutemwa 2006; Soeung *et al.* 2006; Maluka *et al.* 2010, 2011a; Nnaji *et al.* 2010; La Vincente *et al.* 2013) and four to report an acceptable quality (Murthy 1998; de Savigny *et al.* 2008; Maluka *et al.* 2011b; Mutale *et al.* 2013).

### Study characteristics

Table 1 shows the characteristics for the studies in this review including the study location, study design, study participants and the quality assessment level assigned.

**Table 1.** Characteristics of studies of a decision-making process for public health

Article (ID number, author, year)	Location	Study design	Study participants	Study quality^a^
**1. La Vincente *et al.* (2013)**	Philippines	Case study	Regional health office staff (province and city), covering three Local Government Units	+++
**2. Mutale *et al.* (2013)**	Ghana	Case study	Community health officers, District leaders and managers working in public health	++
	Mozambique	Case study	District and provincial health managers, Facility managers and staff	
**3. Maluka *et al.* (2011a)**	Tanzania	Realist evaluation	Government policy makers	+++
**4. Maluka *et al.* (2011b)**	Tanzania	Case study	Administrators, Health Managers, NGO Staff, members of Council Health Management Team (CHMT), Council Health Services Board, district administrative officials, private health service providers, advocacy organizations, knowledgeable community members	++
**5. Maluka *et al.* (2010)**	Tanzania	Case study	Administrators, Health Managers, NGO Staff, Members of FBOs, knowledgeable members of the community	+++
**6. Nnaji *et al.* (2008)**	Nigeria	Case study	District Health Board Chief Executive Officer, Local Health Authority secretaries, Members of DHB	+++
**7. de Savigny *et al.* (2008)**^b^	Tanzania	Case study	District health council management teams	++
**8. Mutemwa (2006)**	Zambia	Case series in 2 district health systems (4 retrospective, 4 concurrent)	District health managers and other members of District Health Management Team or broader district health office	+++
**9. Soeung *et al.* (2006)**	Cambodia	Case study	Health manager, Health centre staff	+++
**10. Chaulagai *et al.* (2005)**	Malawi	Case study	Health Managers, District Health Management Team	+++
**11. Mubyazi *et al.* (2004)**	Tanzania	Case series in 4 districts	District Commissioners, Administrative Secretaries, Medical Officers, Health Secretaries, Treasurers, Hospital Medical Superintendents; Council Executive Directors, Health Officers and Planning Officers; Dispensary and Health centre staff; Village leaders and development committees; Ward Development Committees; Heads of households	+++
**12. Heinonen *et al.* (2000)**	Philippines	Case study	Administrators, Health Managers, General population	+++
**13. Murthy (1998)**	India	Case series in 2 districts	NGO programme officers, District Health Officer, District Family Welfare Officer, Primary health centre staff	++
**14. Sandiford *et al.* (1994)**	Tanzania	Exploratory case study: situation analysis (rapid appraisal & information audit)	Government Policy Makers, Administrators, Health Managers	+++

^a^Level of overall methodological quality of the study adapted from SIGN levels: +++ high quality; ++ acceptable quality, some flaws in the study design; + low quality, significant flaws in the study design.

^b^Additional information found at http://network.idrc.ca/en/ev-56203-201-1-DO_TOPIC.html.

Table 2 shows the characteristics of the health system in each study area to help understand the context in which the decision-making tools were used. Six of the studies took place in countries where decentralization of power in the health system (including budgetary control) was theoretically complete to district level (Sandiford *et al.* 1994; Mutemwa 2006; de Savigny *et al.* 2008; Maluka *et al.* 2010, 2011a). Yet in practice district administrators had limited financial autonomy, affecting their ability to see their decisions through. The rest of the studies took place in countries with limited decentralization and little, or no financial autonomy.

**Table 2.** Characteristics of the health systems in the study areas

Articles (ID number, author, year)	Level of health care	Level of decentralization (for decision-making, authority and power at district level)	Degree of financial autonomy (to set budget and allocate funds accordingly)	Degree of autonomy to move/transfer staff and to allocate non-financial resources
**1. La Vincente *et al.* (2013)**	primary and secondary	Limited	limited	not stated
**2. Mutale *et al.* (2013)**	Ghana: primary	limited	none	not stated
	Mozambique: primary	limited	limited	limited
**3. Maluka *et al.* (2011a)**	primary and secondary	full (taking national planning guidelines into account)	limited	full
**4. Maluka *et al.* (2011b)**	primary and secondary	full (taking national planning guidelines into account)	limited	full
**5. Maluka *et al.* (2010)**	primary and secondary	full (taking national planning guidelines into account)	limited	full
**6. Nnaji *et al.* (2008)**	primary and secondary	Limited	limited	full
**7. de Savigny *et al.* (2008)**	primary	full	limited	full
**8. Mutemwa (2006)**	primary and secondary	full	limited	full
**9. Soeung *et al.* (2006)**	primary	limited (specifically immunization programme and implementation management)	none	limited
**10. Chaulagai *et al.* (2005)**	primary and secondary	limited (in process of gaining autonomy for planning and management of health services)	none	limited
**11. Mubyazi *et al.* (2004)**	primary and secondary	limited	limited	limited (some functions still with central government)
**12. Heinonen *et al.* (2000)**	primary	limited	limited	full
**13. Murthy (1998)**	primary and secondary	limited	none	none
**14. Sandiford *et al.* (1994)**	primary	full	limited	limited

### Evidence-based decision-making processes with set steps and topics addressed

Thirteen of the papers in this literature review outlined the steps involved in the decision-making process used, with three reporting on the same study (Maluka *et al.* 2010, 2011a,b) and one describing two separate decision-making processes (Murthy 1998). Table 3 summarizes these 11 decision-making processes and outlines the topics to which they were applied. The studies described the frameworks or tools used for evidence-based decision-making in health systems at district level, addressing a wide range of different topics. Seven of these studies related to priority setting, annual health planning and budgeting (Murthy 1998; Heinonen *et al.* 2000; Mubyazi *et al.* 2004; Soeung *et al.* 2006; Nnaji *et al.* 2010; Maluka *et al.* 2010, 2011a,b; La Vincente *et al.* 2013), with two of them focussed on decision-making specifically related to maternal and child health, (Murthy 1998; La Vincente *et al.* 2013;) and two based on national agendas (Heinonen *et al.* 2000; Soeung *et al.* 2006). Of these latter two, one was about delivery of immunization services, (Soeung *et al.* 2006) and the other looked at a Minimum Basic Needs (MBN) approach linked to the Government of the Philippines’ national poverty alleviation policy, a broad drive tackling the survival, security and enabling needs of poor people, of which health was just one part (Heinonen *et al.* 2000). MBN aimed to enhance local government autonomy; increase collaboration and coordination between NGOs, people’s organizations and local government; and encourage community members and relevant sectors to participate in planning and project implementation.

**Table 3:** Description of the tool/approach for decision-making (described in 10 studies)^a^

Articles (ID number, author, year)	Themes for decisions	Decision-making framework	Generic steps for decision-making	
**1. La Vincente *et al.* (2013)**	Priority setting; Budget setting by local government units	Investment Case Approach - structured problem solving to identify and develop strategies to overcome key health system problems. A decision-support model estimates cost and impact, guides strategy selection and prioritization	1. **Critical assessment of available evidence** on six parameters related to the performance of health systems	
			2. **Structured, systematic examination of the key constraints** hampering the scaling-up of priority maternal, newborn and child health interventions in disadvantaged populations to identify root cause	
			3. **Identification of feasible strategies to address constraints**, and estimate made of the resultant increases in coverage of relevant health services	
			4. **Estimate of the impact and associated costs of different strategies** made, using an epidemiological and economic decision-support model	
			5. **Review of estimated impact and cost of the different identified strategies**, to guide decision-making for planning and priority setting	
**2. Mutale *et al.* (2013)**	Priority setting and resource allocation	Ghana: District Health Planning and Reporting Toolkit (DiHPART)^b^	1. **Priorities identified**: through overview of local burden of disease profile and its implications for current plans and activities	
			2. **Resource allocation**: through identification of adaptations to align spending priorities with risk patterns	
**3. Maluka *et al.* (2011a)**	Priority setting	Accountability for Reasonableness (A4R) Framework; Core principle: that priority setting decisions should be based on evidence, reasons and principles accepted by stakeholders as relevant to meet health needs fairly in their context. Priority setting process evaluated against A4R: **Relevance; Publicity; Appeals and revision; Enforcement**^c^	1. **Priorities identified** in 6 areas: reproductive and child health; communicable diseases; non-communicable diseases; treatment of common diseases of local priority in districts; community health promotion; strengthening capacity; and organizational structure of health service management; based on local epidemiological data, health service statistics and survey of priorities/needs of hospitals, health centres, dispensaries and the community	
**4. Maluka *et al.* (2011b)**				
			2. **Activities for each priority set for the year**, based on magnitude, severity, feasibility & cost	
**5. Maluka *et al.* (2010)**			3. **Rationale developed for each priority selected**, based on evidence, reasons and principles accepted as relevant by stakeholders	
			4. **Priorities and their rationales made public**	
			5. **Appeals/revisions of decisions made**, in light of new evidence	
			6. **Review of plans and budget** by Council Health Services Board and Full Council, to ensure they meet and address local health priorities	
			7. **Implementation of interventions** (in each priority area, based on magnitudes, severity, feasibility and control at low cost)	
			8. **Monitoring and evaluation** of health service delivery (Community engagement at each stage)	
**6. Nnaji *et al.*(2008)**	Annual budget preparation	Unnamed bottom-up approach for preparing budget estimates for resource allocation	1. **Review** of how community needs, government policies, expected cost effectiveness and distributional impact are met, through pre-budget seminars of programmes, projects and activities	
			2. **Use of one or both expenditure classification systems**: 1) **Functional classification** based on programmes, converted into 2) **Economic classification** (costing) to assist in preparing budget estimates	
			3. **Budget mid-term review**	
			4. **Budget end-term review**	
**8. Mutemwa RI, (2006)**	8 decision-making processes: 6 administrative, 2 epidemiological	No name; 3 stage process	1. **Identification of problem** (understanding the problem situation and identifying the problem to be targeted)	
			2. **Investigation** (information gathering to understand the root cause of the problem and its impact on the organization or services)	
			3. **Solution development** (activities to develop a solution - may be a complex programme or simple list of intervention activities)(not implementation)(Each stage has transitional links but also a distinct set of activities)	
**9. Soeung *et al.* (2006)**	Improvement in coverage of Immunization through micro-planning	Coverage Improvement Planning (CIP) from which micro plans were developed for health centres and villages with local area populations of ∼10 000 per health centre	1. **Mapping** of health centre areas to identify unimmunized children and barriers to improved coverage	
			2. **Identification of solutions** to remove barriers to improved coverage and identify projected costs for reaching coverage goals, in initial workshop for health workers and managers	
			3. **Development of coverage improvement plan** in further workshop	
			4. **Development of budget for plan**	
			5. **Setting of performance agreements** between various government levels	
			6. **Financing and implementation of plan**	
			7. **Monitoring of plan**	
**10. Chaulagai *et al.* (2005)**	Devising tool to improve management and use of health information	No specific named tool for decision-making. Brings all stakeholders together in a workshop and all the decisions are made by understanding and agreeing to the fact the a new improved HMIS is needed which then is devised and introduced	1. **Identification of minimum indicators, datasets and a 5 year strategy** for strengthening the routine HMIS	
			2. **Consensus on indicators** for inclusion	
			3. **Revision of tools** for data collection, processing, reporting and use of information in routine management at local and district level	
			4. **Testing of revised procedures and manuals,** for 18 months in phases (starting with 3 health facilities, then entire district and tertiary care facility)	
			5. **Training of District Health Management Team members** in 6 months using cascade-training approach	
			6. **System implemented** throughout the country from January 2002	
			7. **Revised curricula of pre-service health training programmes**, to include newly devised HMIS tools and procedures	
			8. **Amended job descriptions for health and support staff**, to include information management and use, with regular meetings and reporting	
			9. **Development of tools for annual health sector joint review,** health information policy, indicator handbook, routine monitoring and guidelines	
**11. Mubyazi *et al.* (2004)**	Priority setting in primary and secondary health problems for annual district health plans	Ministry of Health/Ministry of Regional Administration and Local Government Council Planning Guidelines for Health Basket Grant. National essential health package, used to identify local health problems	1. **Identification of health problems**, by Council Health Management Team for district using guidelines	
			2. **Development of comprehensive district health plan** with District Planning Officer and District Treasurer	
			3. **Endorsement of plan** by District Council	
			4. **Review and feedback** at regional level	
			5. **Review and approval** by Ministry of Health before funding is released	
**12. Heinonen *et al.* (2000)**	Linked in to central government poverty alleviation policy, that includes: people’s *Survival, Security* and*Enabling needs*	Minimum Basic Needs Approach (MBN), to enhance local government autonomy, increase collaboration and coordination between NGOs, community based organizations and local government units, and encourage participation of community members and various sectors in planning and project implementation	1. **Formation of MBN team,** by existing inter-agency technical working group convened by local government (MBN includes municipal planners and local representatives from Departments of Health, Agriculture and Social Welfare & Development)	
			2. **Planning and delivery of training on MBN Approach**, to mobilize local health volunteers, community organization leaders and grassroots groups	
			3. **Identification of basic needs not being met,** (out of ∼33) from household data collected by trained volunteers	
			4. **Management and analysis of household data** by community members with assistance from the MBN Team	
			5. **Findings summarized and presented** at a public forum	
			6. **Identification and ranking of unmet needs** by degree of importance according to community’s criteria	
			7. **Planning of interventions and activities** by community members	
**13. Murthy (1998)**	District health planning and implementation to improve maternal care	No names; different decision-making process in each of two districts	District A:	1. **Formation of district planning team**
				2. **Primary data collection** to identify priorities**,** through household survey based on gap between priority goals and achievement levels in Primary Health Centres (PHCs)
				3. **Initiation of planning process**, by District Family Welfare Officer (DFWO)
				4. **Review of performance** in 15/33 below average PHCs
				5. **Development of 6-point action plan**, by PHC staff
				6. **Implementation** on 2 planning points agreed by DFWO
			District B:	1. **Formation of state level steering committee and implementing committee**
				2. **Suggestions for improving services** made by expert group appointed by the government
				3. **Household survey conducted**
				4. **Review of findings** by PHC staff
				5. **District facility survey conducted**
				6. **Decision on focus for district plan**, by Implementation Committee
				7. **Pilot implementation of actions** listed in plan
				8. **Revision of implementation**
				9. **Actions checked against government guidelines**
				10. **Full implementation of actions** listed in plan
				11. **Monitoring of implementation**
				12. **Selection of new problems for action**
**14. Sandiford *et al.* (1994)**	Testing hypothesis: that decentralized decision-making, can improve management of health services through; *Training*, *Elaboration* and use of procedures, and *Development* of improved HMIS	Audit by Issue for Health Management (AIHM); it is based closely on the *District Action Research and Evaluation* process, but differs by employing strict criteria for issue selection and bases decision-making on the information generated through prior analyses of relevant data, derived from routine HMIS or *ad hoc* inquiries.	1. **A priori appraisal** of the scope for management intervention	
			2. **Audit protocol developed, tested and applied** to generate information relevant to the issue	
			3. **Results presented to a meeting of District Health Management Team** where decisions are taken and a detailed action plan agreed	

^a^One study (7. de Savigny *et al.* 2008) did not outline the steps in the decision-making process that was used, so has not been included in this table.

^b^No framework described for Mozambique, where there is a strategy to improve the quality of HMIS data used for district-level decision-making to improve service delivery.

^c^Three papers in this literature review refer to the same study (3. Maluka *et al.* 2010; 4. Maluka *et al.* 2011a; 5. Maluka *et al.* 2011b).

The other four studies were about decision-making on different topics: one helped district managers with health resource allocation based on the local burden of disease profile (Mutale *et al.* 2013); one evaluated eight decision-making processes (four historical and four concurrent with the study), of which six were administrative decisions and two were epidemiological; (Mutemwa 2006) and one tested the hypothesis that in decentralized decision-making, health services management can be improved by employing three training approaches—training health systems’ managers; the elaboration and use of procedures for regular planning and evaluation; and the development of improved HMIS to ensure a good evidence base for decision-making (Sandiford *et al.* 1994). The other study focused on HMIS and the decisions for developing a tool to improve the management and use of health information (Chaulagai *et al.* 2005).

In all, only five of the studies linked the decision-making process used to a system or service outcome (Sandiford *et al.* 1994; Murthy 1998; Mubyazi *et al.* 2004; Chaulagai *et al.* 2005; Soeung *et al.* 2006). None of the studies reported any steps that evaluated effectiveness of the decision-making processes they described.

### Data sources used and their quality

Table 4 shows the various sources of data used in each study. While HMIS is a widely used source of data, (Sandiford *et al.* 1994; Murthy 1998; Mubyazi *et al.* 2004; Chaulagai *et al.* 2005; Mutemwa 2006; de Savigny *et al.* 2008; Maluka *et al.* 2010, 2011a, b; Nnaji *et al.* 2010; La Vincente *et al.* 2013; Mutale *et al.* 2013 (Ghana)) in no case was it the sole source. A few studies highlighted weaknesses in HMIS data (Mubyazi *et al.* 2004; Nnaji *et al.* 2010; Maluka *et al.* 2011a; La Vincente *et al.* 2013); that they were generally produced for national use and had some limitations in local scope, reliability, validity and timeliness. La Vincente *et al.* (2013) considered the lack of local data to be ‘…a major impediment to local planning’, particularly in relation to mortality rates and causes, health service coverage parameters and health system costs.

**Table 4:** Sources of data used for decision-making

Article (ID number, author, date)	HMIS data	Facility records	Document reviews	Other sources of data^a^
**1. La Vincente *et al.* (2013)**	Yes	Limited	Yes	Special surveys and studies
**2. Mutale *et al.* (2013)**	Ghana: Yes	Yes	Yes	No
	Mozambique: No	Yes	No	No
**3. Maluka *et al.* (2011a)**	Yes	Yes	No	Expert opinion (from workshops), National policy requirements, Conducted survey of priorities/needs of hospitals, health centres, dispensaries and community
**4. Maluka *et al.* (2011b)**				
**5. Maluka *et al.* (2010)**				
**6. Nnaji *et al.* (2008)**	Yes	No	Yes	No
**7. de Savigny *et al.* (2008)**	Yes	No	Yes	Demographic Surveillance System
**8. Mutemwa (2006)**	Yes	No	Yes	Observational, Discussion, Experiential (through supervisory visits and consultative visits), Training
**9. Soeung *et al.* (2006)**	No	No	Yes	Data from CIP micro-plan activities; Observational description of introduction of a pilot project
**10. Chaulagai *et al.* (2005)**	Yes	Yes	Yes	Findings from an analysis of strengths and weaknesses of the existing information system
**11. Mubyazi *et al.* (2004)**	Yes	No	Yes	Studies and information collected by vertical programmes, information through community channels
**12. Heinonen *et al.* (2000)**	No	No	No	Conducted household surveys, Focus group discussions, Discussion
**13. Murthy (1998)**	Yes	Yes	No	Conducted household and facility surveys, Observation at mother and child protection camps
**14. Sandiford *et al.* (1994)**	Yes	Yes	Yes	Catchment population estimates

^a^Brief description of other data sources, where applicable.

### Consensus building mechanisms and their effectiveness

Decision-making processes may be considered successful if they help decision makers reach consensus. Over 80% of the studies described decision-making processes with a built-in consensus mechanism (Sandiford *et al.* 1994; Murthy 1998 (District B); Heinonen *et al.* 2000; Mubyazi *et al.* 2004; Chaulagai *et al.* 2005; Mutemwa 2006; Soeung *et al.* 2006; Maluka *et al.* 2011a; La Vincente *et al.* 2013; Mutale *et al.* 2013 (Ghana)), whereas in the rest it was lacking. Consensus decision-making is a way in which common agreement is reached and supported by all members of a group. La Vincente *et al.* (2013) noted problem solving workshops at which stakeholders had structured discussions to develop strategies to overcome constraints in scaling up maternal, newborn and child health interventions. In Zambia, (Mutemwa 2006) a three-stage decision-making process was described, consisting of problem recognition, investigation and solution development, in which the third stage entailed agreeing a list of recommendations for action. In practice, some consensus mechanisms did not function properly; Maluka *et al.* (2010) found an imbalance of power among stakeholders with some participants feeling ill-prepared because of insufficient time or access to documentation; others did not have a full awareness of their role and responsibilities (Maluka *et al.* 2011a); and some felt that decisions were made by the District Medical Officer, without recourse to the agreed decision-making process (Maluka *et al.* 2011b).

### Challenges to decision-making processes

The decision-making processes described above and in Table 3 have not always worked smoothly in practice. This review shows that the challenges fall broadly into three categories; the availability and quality of health and health facility data; human dynamics within a formal, data-based decision-making process; and decisions compromised by financial constraints.

#### 1. Availability, quality and use of health and health facility data

A lack of data available at sub-national level and difficulties in accessing data were reported (La Vincente *et al.* 2013). Some data were found to be unreliable, not produced in a timely manner to contribute to the decision-making process (Maluka *et al.* 2010, 2011a; Nnaji *et al.* 2010). In Tanzania, HMIS data, being centrally defined and geared towards upward reporting, did not allow for the adaptations needed for local planning, moreover data for vertically funded programmes were not always copied to the District Medical Officer (Mubyazi *et al.* 2004). In Nigeria, data from HMIS, the Human Resources Management and Financial Management Systems were not considered reliable (Nnaji *et al.* 2010). Although information on the cost of health system inputs was critical for the development of sound plans, La Vincente *et al.* (2013) found that many costs were not routinely available for Local Government Units to use in planning. In Malawi, when deciding the minimum data set for inclusion in HMIS, reaching consensus was considered a challenge because stakeholders wanted to include all possible indicators for routine collection, including financial, human resources, physical assets and logistical information (Chaulagai *et al.* 2005).

#### 2. Social and political dynamics in the decision-making process

Some concerns were raised that decisions were not always based on data. In one study the decision-making process was derailed, or ‘corrupted’, e.g. due to political conflict, so that no decision was made and the original problem remained unresolved (Mutemwa 2006). Heinonen *et al.* (2000) noted that people who had influence in the community could sway decisions, and thus the needs of some groups were not heard. In one district in India, the intention was to prioritize planning on the issue with the greatest gap between health goals and the level of achievement. However, health programme officers ignored local data and made a decision based on national priorities, workers’ previous achievements and those health care facilities responsible for the majority of antenatal service provision (Murthy 1998 (District A)).

In Tanzania, de Savigny *et al.* (2008) emphasized that tools are necessary but not sufficient, and that capacity strengthening in the form of training to develop management, administration and other skills related to planning and informal mentoring to cultivate a team approach was also needed. Mubyazi *et al.* (2004) suggested a lack of clarity about the conceptualization and operationalization of multisectoral planning and participation, as well as limited capacity to manage the democratic participatory and multidisciplinary processes involved. Inequity in participatory processes was also an issue. Maluka *et al.* (2011b) noted that community members raised concerns that their voices were not heard, and mechanisms for disseminating the priorities identified were found to be ineffective (Maluka *et al.* 2010). Moreover, decision-making processes were seen as dominated by district health professionals on the Council Health Management Team (CHMT), because although the planning guidelines included provision for community and other stakeholder representation, through members of health committees and boards that worked in partnership with the CHMT to provide input for the Comprehensive Council Health Plan, in practice the health committees rarely met (Maluka *et al.* 2010). In addition, there was no mechanism in place to ensure that the community received the plan and only limited opportunities for them to appeal against a decision, both of which were included in the conditions for the Accountability for Reasonableness priority-setting framework (Maluka *et al.* 2011a). In Nigeria too, it was noted that neither community nor private sector representatives were fully involved in decision-making processes (Nnaji *et al.* 2010).

#### 3. Decisions compromised by financial constraints

Local decisions were expected to be made taking account of available funding, but this was not always the case. Four studies noted financial constraints: in Ghana, a lack of flexible funds led to a disconnect between plans and expenditure (Mutale *et al.* 2013). Maluka *et al.* (2011b) also found National Planning Guidelines and budget ceilings limited local level planning and financial allocation, and efforts to engage multiple stakeholders in decision-making process were constrained by delays in the disbursement of funds from central government (Maluka *et al.* 2011a). Heinonen *et al.* (2000) noted that in the Philippines greater financial autonomy had brought with it an expectation that funds would be raised locally, but that securing finance was both competitive and time-consuming, and with limited overall funding, it was easy to lose motivation over time.

## Discussion

We found 12 examples of the implementation of tools to assist a structured process for district level decision-making using local health data, all of which included two key stages—the identification of priorities and the development of an action plan to address them. In eight of these tools HMIS data were used for prioritization. The use of HMIS data at other stages in the process was explicitly stated in one study, which documented three separate cases of HMIS data being used throughout a three-step decision-making process—problem recognition, investigation and solution development—and also gave three cases where HMIS data were used in two of those three steps (Mutemwa 2006). A further four tools identified included a step to review or monitor the action plan agreed, suggesting HMIS data use (Murthy 1998 (District B); Maluka *et al.* 2010, 2011a; Nnaji *et al.* 2010; La Vincente *et al.* 2013).Yet the effectiveness of these formal processes may be limited by various factors, including the poor quality and limited availability of health and health facility data, lack of coordination and capacity among decision makers, and lack of autonomy, (Shaikh *et al.* 2012) which may restrict how closely local health priorities are reflected in service provision. This suggests that for a standardized tool to function properly it needs to be introduced, alongside formal and/or informal support to strengthen the capacity and skills of district level decision makers, (de Savigny *et al.* 2008) within a setting where formal, decentralized authority with the financial autonomy to be able to implement decisions, allows them to make and carry through decisions.

## Limitations

This literature review included peer-reviewed studies in English and French, and grey literature in English. We focused on identifying examples of data being used for decision-making (rather than for any other use, e.g. service coverage) and on any structured decision-making processes, rather than considering other factors that may influence the process, such as the need to work within budgetary or resource constraints; the political will of group members; the amount of time available to assess all the options to be considered and the leadership style within the group. None of the studies we identified outlined any formative work that had been done to develop and test the decision-making process described.

Limitations of the field mean that much of the knowledge generated by NGO projects and interventions is not widely available on the Internet. Therefore, this literature review can only provide a snapshot of activity that has taken place to encourage data used in district-level decision-making.

### Structured decision-making processes

Insights from experts working in the water and energy sectors provide both a definition and an example of a structured decision-making process for a multi-stakeholder group. A structured decision-making process is a set of predefined steps that includes consensus building and incorporates the use of locally generated data, to offer a consistent approach for use in complex situations, through recognizing and understanding the context, and developing and evaluating innovative solutions (Compass Resource Management Ltd. 2013, 2014). To this definition we would add that a structured decision-making process for the health sector would also be replicable within different health systems. Drawing on behavioural decision research, Wilson and Arvai (2011) have developed one such approach, called *Structured Decision Making*, which was designed to improve stakeholder participation, through a series of facilitated steps for analysing the context, evaluating potential solutions and considering trade-offs between possible solutions. Structured Decision Making was predicated on ways to cope with disagreements and reach an agreement that all the stakeholders can own and will then help to implement.

Sandiford *et al.* (1994) described the use of a structured process—Audit by Issue for Health Management (AIHM)—in two decision-making exercises in Kisarawe District, Tanzania using health facility and staffing data: one for staff distribution and one for drug kit distribution. AIHM was a three-step process: an *a priori* appraisal of the scope for management intervention; an audit protocol, tested and applied to generate relevant information and a meeting of the District Health Management Team to discuss and agree an action plan. AIHM was successful in one instance, in that it led to a better distribution of staff at dispensaries in the district, but not in the other instance, due to the team’s limited autonomy, primarily limited financial autonomy, to make decisions about the issue being discussed. Documentation of any subsequent use of AIHM is not available in the public sphere.

### Key features of a decision-making process

After reviewing the data, we found three features that kept recurring: that relevant and good quality data are prerequisite; that a structured decision-making process needs to include characteristics that will help to build consensus; and that the community can to have a well-defined role.

Timely, accurate and relevant health data contributes to decisions that will bring about improvements to the functioning of health facilities and the health system (AbouZahr and Boerma 2005). Yet the relevance and quality of HMIS data in low- and lower-middle-income countries are often compromised and accessing data in a timely manner is usually difficult (Braa *et al.* 2007, 2012). To improve the reliability and comparability of data from different sources HMIS data need to be standardized and harmonized. Useful data relating to the local population and services they use, can be drawn from a range of sectors including health, education, economics and transport, as well as census and national survey data (Stansfield *et al.* 2006). Close interaction between data producers—those who design, implement and manage information systems—(including, but not limited to those producing data for HMIS) and data users are essential if the data are to be relevant, comprehensible and timely for district-level decision makers, as well as those at national level (Nutley 2012).

In reaching consensus, those involved may take ownership of the decisions made, resulting in greater support for implementation (Wilson and Arvai 2011). The inclusion of a facilitated, structured mechanism to build consensus will help to make the decision-making process smooth and efficient. While documented examples of consensus-based decision-making at district level were limited, there were good examples at community level. In a study from Ethiopia, health staff and community members listed and ranked critical maternal and child health behaviours that caused childhood mortality and morbidity; produced a joint action plan to explore these behaviours and the constraints to adopting healthy ones, and strategies to overcome them both, before undertaking joint data analysis of household surveys. At each stage, community meetings to discuss social mapping, free listing, questionnaire development, matrix ranking, constraints and strategies to overcome them, all involved reaching consensus (Bhattacharyya and Murray 2000). Bhattacharyya and Murray (2000) also describe activities to help with a consensus-based decision-making approach, such as training for facilitators to support the process, and training for participants, ‘…to improve their skills in building rapport, listening, and asking open-ended questions’.

Involving the community in the decision-making process helps to identify local health priorities and encourage uptake and monitoring of health services, enhancing a sense of ownership and improving accountability (Israr and Islam 2006).

To these prerequisites and characteristics we add a recommendation that the decision-making process is standardized to help replicability. Operationally, we define standardization as a well-defined protocol for specifying and implementing a process which is intended to be used repeatedly, in order to achieve operational consistency provided the process is repeated in the same context. The ISO (International Organization for Standardization) sees standardization of processes as creating guidelines that can be used consistently to ensure processes are fit for purpose (ISO website). Its guidelines for developing standards for processes recommend collaboration between stakeholders. While the technical aspects of a standardized decision-making process are likely to be broadly the same across the sub-national level, local socio-political priorities within a district will play a part in the interpretation and application of that process. The interplay of such elements should be acknowledged openly to ensure that the process is transparent and decision makers are accountable to their local population. Transparency and accountability are underlying factors for a standardized decision-making process. None of the studies we found mentions using a standardized process or tool for decision-making (i.e. adopting one grounded in a theoretical framework, and for which stakeholders were engaged in the development process, pretesting and pilot testing) which would offer decision makers some methodological assurance of a reliable process for making decisions.

### Challenges to data-based decision-making at district level

It should be possible to overcome the three challenges to formal data-based decision-making for health at district level in low- and lower-middle-income countries that we have identified:

### 1. Limited health system decentralization

Within a centralized health system district-level decision makers have no autonomy to make and implement decisions to improve local health outcomes. Yet where there has been decentralization, the level of autonomy that the decision makers have over resources depends largely on the type of decentralization that has been introduced and whether it includes financial autonomy to put decisions into practice. In an analysis of decision space, Bossert *et al.* compared decision-making in decentralized health systems in four countries with four different types of autonomy: deconcentration, devolution, delegation and privatization (Bossert and Beauvais 2002). Of these, devolution, gave district level decision makers in the Philippines a greater degree of autonomy over finances, service organization, human resources and governance, because of their apparent fiscal and administrative capacity. In comparison, when authority was delegated to semi-autonomous agencies in the Ghanaian health system, autonomy over finances, resources and management was compromised. Decentralization through devolution theoretically gives decision makers full financial and administrative autonomy, yet where this has not happened in practice, as is often the case, the benefits are more limited (Somanje *et al.* 2012).

### 2. Quality and availability of health data for district-level use

We mentioned above the need for timely, accurate and relevant data for decentralized decision making. Local health data are usually collected for amalgamation into national HMIS from which reports are created largely for central use, but which may also be cascaded back to district level. This process takes time and therefore local utilization of the data at the point of collection is delayed and data may have been superseded in relevance. Moreover, the granularity of the data is lost in national reports, thus compromising the detail required by local users. Harrison and Nutley (2010) suggest that reasons for problems with data accuracy, completeness and quality include complex procedures for reporting data; limited access to computers to record data digitally and the prevalence of inaccuracies made through manual recording; and the limited time available to health facility staff to compile data.

### 3. Capacity development of decision makers to use data

Alongside the need improve the quality and availability of health data, there is also the challenge of district health officers often having limited capacity to understand and utilize it for decision-making. This could be addressed in part by equipping the stakeholders involved with the knowledge and skills to do so. For example, a recent study in Kenya showed the positive impact on improving and planning health services of a decision support tool for aggregating, analysing and presenting data in a faster and more simplified format (Nutley *et al.* 2013). The use of a structured process can not only help decision makers make priority decisions, but can also increase the demand for, and the availability and quality of data.

## Conclusion

We found a number of examples of decision-making processes that include the use of local HMIS data, yet there was limited evidence about their sustained impact on district level decision-making and whether they have led to changes in resource allocation patterns. Operational research could reveal adaptations needed for a variety of local contexts and if research was undertaken to assess whether their use had brought about revisions in the allocation of resources. In addition, we found no information of steps taken to create and pilot these decision-making processes. Such information would contribute to their standardization. Further research evidence in these areas would help to address the limitations of the current body of evidence.

## Supplementary Material

Supplementary Data
