# Supplementary material for: District decision-making for health in low-income settings: a systematic literature review
Source: Health Policy Plan. 2016 Sep 1;31(Suppl 2):ii12–24. doi: 10.1093/heapol/czv124 (PMC5009221; doi:10.1093/heapol/czv124)
Supplement: Supplementary Data [file supp_31_suppl-2_ii12__index.html]

District decision-making for health in low-income settings: a systematic literature review — Supplementary Data 

# District decision-making for health in low-income settings: a systematic literature review

## Supplementary Data

files

- Supplementary Data - zip file
