# Supplementary material for: District decision-making for health in low-income settings: a systematic literature review
Source: Health Policy Plan. 2016 Sep 1;31(Suppl 2):ii12–24. doi: 10.1093/heapol/czv124 (PMC5009221; doi:10.1093/heapol/czv124)
Supplement: Supplementary Data [file supp_czv124_suppl_data.zip › DistrictDecisionMaking_Paper2_Table2.docx]

**Table 2: Characteristics of the health systems in the study areas**

| **Articles (ID number, author, year)** | **Level of health care** | **Level of decentralisation (for decision-making, authority and power at district level)** | **Degree of financial autonomy (to set budget and allocate funds accordingly)** | **Degree of autonomy to move/transfer staff and to allocate non-financial resources** |
| --- | --- | --- | --- | --- |
| 1. **La Vincente S, et al (2013)** | primary and secondary | Limited | limited | not stated |
| 1. **Mutale W, et al (2013)** | Ghana: primary | limited | none | not stated |
|  | Mozambique: primary | limited | limited | limited |
| 1. **Maluka S, et al (2011a)** | primary and secondary | full (taking national planning guidelines into account) | limited | full |
| 1. **Maluka S, et al (2011b)** | primary and secondary | full (taking national planning guidelines into account) | limited | full |
| 1. **Maluka S, et al (2010)** | primary and secondary | full (taking national planning guidelines into account) | limited | full |
| 1. **Nnaji GA, et al (2008)** | primary and secondary | Limited | limited | full |
| 1. **de Savigny D, et al (2008)** | primary | full | limited | full |
| 1. **Mutemwa RI, (2006)** | primary and secondary | full | limited | full |
| 1. **Soeung SC, et al (2006)** | primary | limited (specifically immunisation programme and implementation management) | none | limited |
| 1. **Chaulagai CN, et al (2005)** | primary and secondary | limited (in process of gaining autonomy for planning and management of health services) | none | limited |
| 1. **Mubyazi G, et al (2004)** | primary and secondary | limited | limited | limited (some functions still with central government) |
| 1. **Heinonen T, et al (2000)** | primary | limited | limited | full |
| 1. **Murthy N, (1998)** | primary and secondary | limited | none | none |
| 1. **Sandiford P, et al (1994)** | primary | full | limited | limited |
