# Supplementary material for: District decision-making for health in low-income settings: a systematic literature review
Source: Health Policy Plan. 2016 Sep 1;31(Suppl 2):ii12–24. doi: 10.1093/heapol/czv124 (PMC5009221; doi:10.1093/heapol/czv124)
Supplement: Supplementary Data [file supp_czv124_suppl_data.zip › DistrictDecisionMaking_Paper2_Table3.docx]

**Table 3: Description of the tool/ approach for decision-making (described in 10 studies)^*^**

| **Articles (ID number, author, year)** | **Themes for decisions** | **Decision-making framework** | **Generic steps for decision-making** | |
| --- | --- | --- | --- | --- |
| **1. La Vincente S, et al (2013)** | Priority setting;  Budget setting by local government units | Investment Case Approach - structured problem solving to identify and develop strategies to overcome key health system problems. A decision-support model estimates cost and impact, guides strategy selection and prioritisation. | 1. **Critical assessment of available evidence** on six parameters related to the performance of health systems | |
|  |  |  | 2. **Structured, systematic examination of the key constraints** hampering the scaling-up of priority maternal, newborn and child health interventions in disadvantaged populations to identify root cause | |
|  |  |  | 3**. Identification of feasible strategies to address constraints**, and estimate made of the resultant increases in coverage of relevant health services | |
|  |  |  | 4. **Estimate of the impact and associated costs of different strategies** made, using an epidemiological and economic decision-support model | |
|  |  |  | 5. **Review of estimated impact and cost of the different identified strategies**, to guide decision-making for planning and priority setting | |
| **2. Mutale W, et al (2013)** | Priority setting and resource allocation | # Ghana: District Health Planning and Reporting Toolkit (DiHPART) | 1. **Priorities identified**: through overview of local burden of disease profile and its implications for current plans and activities | |
|  |  |  | 2. **Resource allocation**: through identification of adaptations to align spending priorities with risk patterns | |
| **3. Maluka S, et al (2011a)**  **4. Maluka S, et al (2011b)**  **5. Maluka S, et al (2010)** | Priority setting | ~ Accountability for Reasonableness (A4R) Framework; Core principle: that priority setting decisions should be based on evidence, reasons and principles accepted by stakeholders as relevant to meet health needs fairly in their context. Priority setting process evaluated against A4R:  **Relevance; Publicity; Appeals & revision;**  **Enforcement** | 1. **Priorities identified** in 6 areas: reproductive and child health; communicable diseases; non-communicable diseases; treatment of common diseases of local priority in districts; community health promotion; strengthening capacity; and organisational structure of health service management; based on local epidemiological data, health service statistics and survey of priorities/needs of hospitals, health centres, dispensaries and the community | |
|  |  |  | 2. **Activities for each priority set for the year**, based on magnitude, severity, feasibility & cost | |
|  |  |  | 3. **Rationale developed for each priority selected**, based on evidence, reasons and principles accepted as relevant by stakeholders | |
|  |  |  | 4. **Priorities and their rationales made public** | |
|  |  |  | 5. **Appeals/revisions of decisions made**, in light of new evidence | |
|  |  |  | 6. **Review of plans and budget** by Council Health Services Board and Full Council, to ensure they meet and address local health priorities | |
|  |  |  | 7. **Implementation of interventions** (in each priority area, based on magnitudes, severity, feasibility and control at low cost) | |
|  |  |  | 8. **Monitoring and evaluation** of health service delivery (Community engagement at each stage) | |
| **6. Nnaji GA, et al (2008)** | Annual budget preparation | Unnamed bottom-up approach for preparing budget estimates for resource allocation | 1. **Review** of how community needs, government policies, expected cost effectiveness and distributional impact are met, through pre-budget seminars of programmes, projects and activities | |
|  |  |  | 2. **Use of one or both expenditure classification systems**: 1) **Functional classification** based on programmes, converted into 2) **Economic classification** (costing) to assist in preparing budget estimates | |
|  |  |  | 3. **Budget mid-term review** | |
|  |  |  | 4. **Budget end-term review** | |
| **8. Mutemwa RI, (2006)** | 8 decision-making processes:  6 administrative,  2 epidemiological | No name; 3 stage process | 1. **Identification of problem** (understanding the problem situation and identifying the problem to be targeted) | |
|  |  |  | 2. **Investigation** (information gathering to understand the root cause of the problem and its impact on the organisation or services) | |
|  |  |  | 3. **Solution development** (activities to develop a solution - may be a complex programme or simple list of intervention activities)(not implementation)  (Each stage has transitional links but also a distinct set of activities) | |
| **9. Soeung SC, et al (2006)** | Improvement in coverage of Immunisation through micro-planning | Coverage Improvement Planning (CIP) from which micro plans were developed for health centres and villages with local area populations of approximately 10,000 per health centre | 1. **Mapping** of health centre areas to identify unimmunised children and barriers to improved coverage | |
|  |  |  | 2. **Identification of solutions** to remove barriers to improved coverage and identify projected costs for reaching coverage goals, in initial workshop for health workers and managers | |
|  |  |  | 3. **Development of coverage improvement plan** in further workshop | |
|  |  |  | 4. **Development of budget for plan** | |
|  |  |  | 5. **Setting of performance agreements** between various government levels | |
|  |  |  | 6. **Financing and implementation of plan** | |
|  |  |  | 7. **Monitoring of plan** | |
| **10.Chaulagai CN, et al (2005)** | Devising tool to improve management and use of health information | No specific named tool for decision-making. Brings all stakeholders together in a workshop and all the decisions are made by understanding and agreeing to the fact the a new improved HMIS is needed which then is devised and introduced | 1. **Identification of minimum indicators, datasets and a 5 year strategy** for strengthening the routine HMIS | |
|  |  |  | 2. **Consensus on indicators** for inclusion | |
|  |  |  | 3. **Revision of tools** for data collection, processing, reporting and use of information in routine management at local and district level | |
|  |  |  | 4. **Testing of revised procedures and manuals,** for 18 months in phases (starting with 3 health facilities, then entire district and tertiary care facility) | |
|  |  |  | 5. **Training of District Health Management Team members** in 6 months using cascade-training approach | |
|  |  |  | 6. **System implemented** throughout the country from January 2002 | |
|  |  |  | 7. **Revised curricula of pre-service health training programmes**, to include newly devised HMIS tools and procedures | |
|  |  |  | 8. **Amended job descriptions for health and support staff**, to include information management and use, with regular meetings and reporting | |
|  |  |  | 9. **Development of tools for annual health sector joint review,** health information policy, indicator handbook, routine monitoring and guidelines | |
| **Articles (ID, author, Year)** | **Themes for decisions** | **Description of the decision-making framework** | **Generic steps for decision-making** | |
| **11. Mubyazi G, et al (2004)** | Priority setting in primary and secondary health problems for annual district health plans | Ministry of Health/ Ministry of Regional Administration and Local Government Council Planning Guidelines for Health Basket Grant. National essential health package, used to identify local health problems. | 1. **Identification of health problems**, by Council Health Management Team for district using guidelines | |
|  |  |  | 2. **Development of comprehensive district health plan** with District Planning Officer and District Treasurer | |
|  |  |  | 3. **Endorsement of plan** by District Council | |
|  |  |  | 4. **Review and feedback** at regional level | |
|  |  |  | 5. **Review and approval** by Ministry of Health before funding is released | |
| **12. Heinonen T, et al (2000)** | Linked in to central government poverty alleviation policy, that includes: people’s *Survival, Security* and *Enabling needs* | Minimum Basic Needs Approach (MBN), to enhance local government autonomy, increase collaboration and coordination between NGOs, community based organisations and local government units, and encourage participation of community members and various sectors in planning and project implementation | 1. **Formation of MBN team,** by existing inter-agency technical working group convened by local government (MBN includes municipal planners and local representatives from Departments of Health, Agriculture and Social Welfare & Development) | |
|  |  |  | 2.**Planning and delivery of training on MBN Approach**, to mobilise local health volunteers, community organisation leaders and grassroots groups | |
|  |  |  | 3. **Identification of basic needs not being met,** (out of approx. 33) from household data collected by trained volunteers | |
|  |  |  | 4. **Management and analysis of household data** by community members with assistance from the MBN Team | |
|  |  |  | 5. **Findings summarised and presented** at a public forum | |
|  |  |  | 6. **Identification and ranking of unmet needs** by degree of importance according to community’s criteria | |
|  |  |  | 7. **Planning of interventions and activities** by community members | |
| **13 Murthy N., (1998)** | District health planning and implementation to improve maternal care | No names; different decision-making process in each of two districts | District A: | 1. **Formation of district planning team** |
|  |  |  |  | 2. **Primary data collection** to identify priorities**,** through household survey based on gap between priority goals and achievement levels in Primary Health Centres (PHCs) |
|  |  |  |  | 3. **Initiation of planning process**, by District Family Welfare Officer (DFWO) |
|  |  |  |  | 4. **Review of performance** in 15/33 below average PHCs |
|  |  |  |  | 5. **Development of 6-point action plan**, by PHC staff |
|  |  |  |  | 6. **Implementation** on 2 planning points agreed by DFWO |
|  |  |  | District B: | 1. **Formation of state level steering committee and implementing committee** |
|  |  |  |  | 2. **Suggestions for improving services** made by expert group appointed by the government |
|  |  |  |  | 3. **Household survey conducted** |
|  |  |  |  | 4. **Review of findings** by PHC staff |
|  |  |  |  | 5. **District facility survey conducted** |
|  |  |  |  | 6. **Decision on focus for district plan**, by Implementation Committee |
|  |  |  |  | 7. **Pilot implementation of actions** listed in plan |
|  |  |  |  | 8. **Revision of implementation** |
|  |  |  |  | 9. **Actions checked against government guidelines** |
|  |  |  |  | 10. **Full implementation of actions** listed in plan |
|  |  |  |  | 11. **Monitoring of implementation** |
|  |  |  |  | 12. **Selection of new problems for action** |
| **14. Sandiford P, et al (1994)** | Testing hypothesis: that decentralised decision-making, can improve management of health services through; *Training*, *Elaboration* and use of procedures, and *Development* of improved HMIS. | Audit by Issue for Health Management (AIHM); it is based closely on the *District Action Research and Evaluation* process, but differs by employing strict criteria for issue selection and bases decision-making on the information generated through prior analyses of relevant data, derived from routine HMIS or *ad hoc* inquiries. | 1. **A priori appraisal** of the scope for management intervention | |
|  |  |  | 2. **Audit protocol developed, tested and applied** to generate information relevant to the issue | |
|  |  |  | 3. **Results presented to a meeting of District Health Management Team** where decisions are taken and a detailed action plan agreed | |

#No framework described for Mozambique, where there is a strategy to improve the quality of HMIS data used for district-level decision-making to improve service delivery.

~ Three papers in this literature review refer to the same study (3. *Maluka et al, 2010; 4. Maluka et al, 2011a; 5. Maluka et al, 2011b*).

* One study (7. *de Savigny* *et al*) did not outline the steps in the decision-making process that was used, so has not been included in this table.
