# Supplementary material for: District decision-making for health in low-income settings: a systematic literature review
Source: Health Policy Plan. 2016 Sep 1;31(Suppl 2):ii12–24. doi: 10.1093/heapol/czv124 (PMC5009221; doi:10.1093/heapol/czv124)
Supplement: Supplementary Data [file supp_czv124_suppl_data.zip › DistrictDecisionMaking_Paper2_SupplementaryFile1.docx]

**Supplementary Data File 1: Search strategy for MEDLINE**

1. decision support techniques/ (10891)

2. Decision Making/ (64141)

3. Decision Making, Organizational/ (10297)

4. consensus/ (4316)

5. Health Planning/ (20312)

6. Community Health Planning/ (4314)

7. Health Systems Plans/ (115)

8. Health Facility Planning/ (1832)

9. Planning Techniques/ (13460)

10. Health planning guidelines/ (3771)

11. Health planning technical assistance/ (215)

12. (decision* or decision-mak* or consensus or (health adj2 plan*) or choice* or assessment or priorit* or strategic planning or decision-space).mp (1313021)

13. 1 or 2 or 3 or 4 or 5 or 6 or 7 or 8 or 9 or 10 or 11 or 12 (1321886)

14. Evidence-Based Practice/ (3421)

15. Health Services Research/ (30042)

16. Decision Support Systems, Management/ (878)

17. Management information systems/ (3575)

18. (evidence or evidence-base* or evidence-informed or evaluation or (knowledge adj1 (use* or usage or utili?ation or transfer or translation or exchange)) or ((data or information) adj1 (use* or usage or utili?ation or exchange)) or information management or information system* or ((programme or project) adj1 (data or information))).mp (2097199)

19. 14 or 15 or 16 or 17 or 18 (2118640)

20. Local government/ (2283)

21. (district* or zone* or woreda* or LGA* or local government or local administ* or local planning or decentrali* or regionali?ation).mp (186745)

22. 20 or 21 (186745)

23. Health care reform/ (26697)

24. health facility administrators/ (4236)

25. Health services administration/ (3911)

26. ((Healthcare adj1 (planning or management or policy or system* or reform)) or (health adj2 (reform or policy)) or health system* or health service* or public health or resource allocation).mp (535368)

27. 23 or 24 or 25 or 26 (538714)

28. (Low-income countr* or developing countr* or (Afghanistan or Bangladesh or Benin or Burkina Faso or Burundi or Cambodia or Central African Republic or Chad or Comoros or Congo or Eritrea or Ethiopia or Gambia or Guinea or Guinea-Bissau or Haiti or Kenya or Korea or Kyrgyz Republic or Liberia or Madagascar or Malawi or Mali or Mauritania or Mozambique or Myanmar or Nepal or Niger or Rwanda or Sierra Leone or Somalia or Tajikistan or Tanzania or Togo or Uganda or Zimbabwe)).mp (351212)

29. (lower-middle-income countr* or LIM or middle-income countr* or (Albania or Armenia or Belize or Bhutan or Bolivia or Cameroon or Cape Verde or Congo or Cote d?Ivoire or Djibouti or Egypt or El Salvador or Fiji or Georgia or Ghana or Guatemala or Guyana or Honduras or Indonesia or India or Iraq or Kiribati or Kosovo or Lao or Lesotho or Marshall Islands or Micronesia or Moldova or Mongolia or Morocco or Nicaragua or Nigeria or Pakistan or Papua New Guinea or Paraguay or Philippines or Samoa or (Sao Tome and Principe) or Senegal or Solomon Islands or Sudan or Sri Lanka or Sudan or Swaziland or Syria* or Timor-Leste or Tonga or Ukraine or Uzbekistan or Vanuatu or Vietnam or Gaza or Yemen or Zambia)).mp (272659)

30. 28 or 29 (575849)

31. 13 and 19 and 22 and 27 and 30 (741)
