# Supplementary material for: District decision-making for health in low-income settings: a systematic literature review
Source: Health Policy Plan. 2016 Sep 1;31(Suppl 2):ii12–24. doi: 10.1093/heapol/czv124 (PMC5009221; doi:10.1093/heapol/czv124)
Supplement: Supplementary Data [file supp_czv124_suppl_data.zip › DistrictDecisionMaking_Paper2_SupplementaryFile2FINAL.docx]

**Supplementary file 2: Data extraction form**

| **Publication details** | |
| --- | --- |
| 1 | ID No. (automatically generated) |
| 2 | Author(s) |
| 3 | Title |
| 4 | Publication date |
| 5 | Journal/ website details |
| 6 | Country/countries |
| 7 | Designation / professional level of decision makers (Health facility, District, Zone, State, Region, National) |
| 8 | Stakeholders in the decision-making process/ Decision makers |
| 9 | Paper included (Y/N/Unsure) |
| 10 | If excluded or unsure, list reasons why |
| **Focus of study** | |
| 11 | Timeframe of data collection for study |
| 12 | Timeframe of intervention |
| 13 | Study informants (government policy makers, administrators, health managers, scientists, NGO staff, etc.) |
| 14 | Type of health sector (Public or government / Private not-for-profit/ Private for profit / Public-Private partnership / other) |
| 15 | List data sources (Lit. Review, interviews, survey, focus groups, observation, mixed methods, etc.) |
| 16 | Thematic area(s) under consideration for decision-making |
| 17 | Additional comments |
| **Literature review themes** | |
| Instruments or tools used for decision-making | |
| 18 | Name and brief description of the decision-making tool (operationalised framework) |
| 19 | Is the tool: new/ pre-existing/ don’t know? (New = never been used before in any form; Pre-existing = includes adapted tools) |
| 20 | List steps used to develop the tool |
| 21 | Properties of the tool |
| 22 | Context in which the tool was used (click as many as applicable) (District/Health facility/Administrative/Budgetary/Human Resource related/Other – please state) |
| Process used for decision-making | |
| 23 | List the sequential steps used by district level decision makers to make decisions |
| 24 | Was consensus built into the decision-making tool, or did the decision makers need to reach consensus? |
| 25 | Is there evidence that the decision-making process used was effective in enabling a consensus to be reached? (yes/no/partially) |
| 26 | Was there evidence that the decision-making tool had included a step to evaluate the effectiveness of the process? |
| Data or evidence used in decision-making | |
| 27 | List type(s) of data used in decision-making |
| 28 | Frequency of decision-making |
| 29 | Frequency of data collection |
| 30 | How the data/evidence are presented to the decision makers (oral presentation at a workshop or meeting/ email/ printed format) |
| Decisions | |
| 31 | What changes in health service delivery resulted from decisions made using the tool? |
| Other secondary questions | |
| 32 | Is the health system where the decision was made decentralised at district level? (yes/no/partially) |
| 33 | Do the decision makers have economic or financial autonomy (control over spending)? |
| 34 | Impact of autonomy on their ability to make decisions (were decision makers able to divert funds?) |
| 35 | What are the challenges or obstacles to an evidence-based decision-making process (numbered list) |
| 36 | Take home message from this study |
