# Supplementary material for: District decision-making for health in low-income settings: a systematic literature review
Source: Health Policy Plan. 2016 Sep 1;31(Suppl 2):ii12–24. doi: 10.1093/heapol/czv124 (PMC5009221; doi:10.1093/heapol/czv124)
Supplement: Supplementary Data [file supp_czv124_suppl_data.zip › DistrictDecisionMaking_Paper2_SupplementaryFile3.docx]

**Supplementary file 3: References for studies included in the literature review with ID number for analysis**

1. La Vincente S, Aldaba B, Firth S, *et al.* 2013. Supporting local planning and budgeting for maternal, neonatal and child health in the Philippines. *Health Research Policy and Systems* **11**:3.
2. Mutale W, Chintu N, Amoroso C, *et al.* 2013. Improving health information systems for decision making across five sub-Saharan African countries: Implementation strategies from the African Health Initiative*. BMC Health Services Research* **13**(Suppl 2):59.
3. Maluka S, Kamuzora P, SanSebastian M, *et al.* 2011(a). Implementing accountability for reasonableness framework at district level in Tanzania: a realist evaluation. *Implementation Science* **6**.
4. Maluka SO, Hurtig A-K, SanSebastian M, *et al.* 2011(b). Decentralization and health care prioritization process in Tanzania: from national rhetoric to local reality. *International Journal of Health Planning and Management* **26**:e102-120.
5. Maluka S, Kamuzora P, San Sebastian M, *et al.* 2010. Decentralized health care priority-setting in Tanzania: Evaluating against the accountability for reasonableness framework. *Socical Science & Medicine* **71**:751-759.
6. Nnaji GA, Oguoma C, Nnaji LI, Nwobodo E. 2010. The challenges of budgeting in a newly introduced district health system: a case study. *Global Public Health* **5**:87-101.
7. de Savigny D, Kasale H, Mbuya C, Reid G. 2004. *Fixing health systems.* 2^nd^ edn. Ottawa: International Development Research Centre.
8. Mutemwa RI. 2006. HMIS and decision-making in Zambia: Re-thinking information solutions for district health management in decentralized health systems. *Health Policy and Planning* **21**:40-52.
9. Soeung SC, Grundy J, Ly CK, *et al.* 2006. Improving immunization coverage through budgeted microplans and sub-national performance agreements: Early experience from Cambodia. *Asia Pacific Journal of Public Health* **18**: 29-38.
10. Chaulagai CN, Moyo CM, Koot J, *et al.* 2005. Design and implementation of a health management information system in Malawi: issues, innovations and results. *Health Policy and Planning* **20**:375-384.
11. Mubyazi G, Kamugisha M, Mushi A, Blas E. 2004. Implications of decentralization for the control of tropical diseases in Tanzania: a case study of four districts. *International Journal of Health Planning and Management.* **19**(Suppl 1):S167-85.
12. Heinonen T, Mercader M, Quianzon JL, Penera-Torralba M, Baluis L. 2000. Conceptualizing and applying a minimum basic needs approach in southern Philippines. *Health Policy and Planning* **15**:194-206.
13. Murthy N. 1998. Decentralized health planning: lessons from two districts in India. *Journal of Health and Population in Developing Countries* **1**:1-10.
14. Sandiford P, Kanga GJ, Ahmed AM. 1994. The management of health services in Tanzania: A plea for health sector reform. *International Journal of Health Planning and Management* **9**:295-308.
