# Supplementary material for: District decision-making for health in low-income settings: a systematic literature review
Source: Health Policy Plan. 2016 Sep 1;31(Suppl 2):ii12–24. doi: 10.1093/heapol/czv124 (PMC5009221; doi:10.1093/heapol/czv124)
Supplement: Supplementary Data [file supp_czv124_suppl_data.zip › DistrictDecisionMaking_Paper2_SupplementaryFile4.docx]

**Supplementary file 4: Results of quality assessment**

|  | **Article ID number *** | **1** | **2** | **3** | **4** | **5** | **6** | **7** | **8** | **9** | **10** | **11** | **12** | **13** | **14** |
| --- | --- | --- | --- | --- | --- | --- | --- | --- | --- | --- | --- | --- | --- | --- | --- |
| **I. Abstract & Introduction** | **a) Is the abstract an informed and balanced summary?** |  |  |  |  |  |  |  |  |  |  |  |  |  |  |
|  | **b) Has the rationale for study explained?** |  |  |  |  |  |  |  |  |  |  |  |  |  |  |
|  | **c) Are the aims and objectives clearly stated?** |  |  |  |  |  |  |  |  |  |  |  |  |  |  |
| **II. Design** | **a) Is the research methodology appropriate to answer the research question?** |  |  |  |  |  |  |  |  |  |  |  |  |  |  |
|  | **b) Have the ethical issues been taken into consideration?** |  |  |  |  |  |  |  |  |  |  |  |  |  |  |
|  | **c) Were the methods employed to collect data/ information appropriate?** |  |  |  |  |  |  |  |  |  |  |  |  |  |  |
| **III. Methodology** | **a) Are the dates (or time period) for data collection mentioned?** |  |  |  |  |  |  |  |  |  |  |  |  |  |  |
|  | **b) Was the selection of participants appropriate to answer the research question?** |  |  |  |  |  |  |  |  |  |  |  |  |  |  |
|  | **c) Has the relationship between researcher and participants been adequately considered?** |  |  |  |  |  |  |  |  |  |  |  |  |  |  |
| **IV. Results** | **a) Is the data analysis sufficiently rigorous?** |  |  |  |  |  |  |  |  |  |  |  |  |  |  |
|  | **b) Are the findings relevant to the research question?** |  |  |  |  |  |  |  |  |  |  |  |  |  |  |
|  | **c) Is there a clear statement of findings?** |  |  |  |  |  |  |  |  |  |  |  |  |  |  |
| **V. Discussion** | **a) Is the conclusion based on the study findings?** |  |  |  |  |  |  |  |  |  |  |  |  |  |  |
|  | **b) Are the limitations of study reported?** |  |  |  |  |  |  |  |  |  |  |  |  |  |  |
|  | **c) Are the implications of the study stated?** |  |  |  |  |  |  |  |  |  |  |  |  |  |  |
| **Total criteria met and level of overall quality of methodology**** | | 12 +++ | 10 ++ | 15 +++ | 11 ++ | 13 +++ | 12 +++ | 8  ++ | 13 +++ | 12 +++ | 13 +++ | 13 +++ | 14 +++ | 11 ++ | 12 +++ |

= Yes = No

* See Supplementary file 3 for a list of the studies in this review with its corresponding Article ID number

** Level of overall methodological quality of the study adapted from SIGN levels: +++ high quality, (meets more than 75% of the criteria); ++ acceptable quality, some flaws in the study design (meets 50-75% of the criteria); + low quality, significant flaws in the study design (meets less than 50% of the criteria)

Cells of the table and *their explanation*:

1. Abstract & Introduction
   1. Is the abstract an informed and balanced summary?
   2. Has the rationale for study explained?
   3. Are the aims and objectives clearly stated?
2. Design
   1. Was the qualitative approach appropriate to answer the research question?
   2. Have the ethical issues been taken into consideration?
   3. Were the methods employed to collect data/information appropriate? *Methods: interviews, focus group discussions, document reviews, HMIS data etc.*
3. Methodology
   1. Were the dates (or time period) for data collection mentioned?
   2. Was the selection of participants appropriate to answer the research question?  *If the researcher has explained why the participants they selected were the most appropriate to provide access to the type of knowledge sought by the study e.g. were the participants involved or affecting the decision-making process in any way at any level?*
   3. Has the relationship between researcher and participants been adequately considered? *If the researcher(s) critically examined their own role, potential bias and influence during collection of information or data*
4. Results
   1. Was the data analysis sufficiently rigorous? *If there is description of analysis process, how the data presented were supporting the findings, and if triangulation has been done.*
   2. Were the findings relevant to the research question?
   3. Was there a clear statement of findings? *Explicit adequate discussion of the findings/evidence.*
5. Discussion
   1. Is the conclusion based on study findings?
   2. Are the limitations of study reported?
   3. Are the implications of study stated?
