# Supplementary material for: District decision-making for health in low-income settings: a systematic literature review
Source: Health Policy Plan. 2016 Sep 1;31(Suppl 2):ii12–24. doi: 10.1093/heapol/czv124 (PMC5009221; doi:10.1093/heapol/czv124)
Supplement: Supplementary Data [file supp_czv124_suppl_data.zip › DistrictDecisionMaking_Paper2_Table1.docx]

**Table 1. Characteristics of studies of a decision-making process for public health**

| **Article (ID number, author, year)** | **Location** | **Study design** | **Study participants** | **Study quality^1^** |
| --- | --- | --- | --- | --- |
| 1. **La Vincente S, et al (2013)** | Philippines | Case study | Regional health office staff (province and city), covering three Local Government Units | +++ |
| 1. **Mutale W, et al (2013)** | Ghana | Case study | Community health officers, District leaders and managers working in public health | ++ |
|  | Mozambique | Case study | District and provincial health managers, Facility managers and staff |  |
| 1. **Maluka S, et al (2011a)** | Tanzania | Realist evaluation | Government policy makers | +++ |
| 1. **Maluka S, et al (2011b)** | Tanzania | Case study | Administrators, Health Managers, NGO Staff, members of Council Health Management Team (CHMT), Council Health Services Board, district administrative officials, private health service providers, advocacy organisations, knowledgeable community members | ++ |
| 1. **Maluka S, et al (2010)** | Tanzania | Case study | Administrators, Health Managers, NGO Staff, Members of FBOs, knowledgeable members of the community | +++ |
| 1. **Nnaji GA, et al (2008)** | Nigeria | Case study | District Health Board Chief Executive Officer, Local Health Authority secretaries, Members of DHB | +++ |
| 1. **de Savigny D, et al (2008) *** | Tanzania | Case study | District health council management teams | ++ |
| 1. **Mutemwa RI, (2006)** | Zambia | Case series in 2 district health systems (4 retrospective, 4 concurrent) | District health managers and other members of District Health Management Team or broader district health office | +++ |
| 1. **Soeung SC, et al (2006)** | Cambodia | Case study | Health manager, Health centre staff | +++ |
| 1. **Chaulagai CN, et al (2005)** | Malawi | Case study | Health Managers, District Health Management Team | +++ |
| 1. **Mubyazi G, et al (2004)** | Tanzania | Case series in 4 districts | District Commissioners, Administrative Secretaries, Medical Officers, Health Secretaries, Treasurers, Hospital Medical Superintendents; Council Executive Directors, Health Officers and Planning Officers; Dispensary and Health centre staff; Village leaders and development committees; Ward Development Committees; Heads of households | +++ |
| 1. **Heinonen T, et al (2000)** | Philippines | Case study | Administrators, Health Managers, General population | +++ |
| 1. **Murthy N, (1998)** | India | Case series in 2 districts | NGO programme officers, District Health Officer, District Family Welfare Officer, Primary health centre staff | ++ |
| 1. **Sandiford P, et al (1994)** | Tanzania | Exploratory case study: situation analysis (rapid appraisal & information audit) | Government Policy Makers, Administrators, Health Managers | +++ |

^*^ Additional information found at http://network.idrc.ca/en/ev-56203-201-1-DO_TOPIC.html

^1^ Level of overall methodological quality of the study adapted from SIGN levels: +++ high quality; ++ acceptable quality, some flaws in the study design; + low quality, significant flaws in the study design
