# Supplementary material for: District decision-making for health in low-income settings: a systematic literature review
Source: Health Policy Plan. 2016 Sep 1;31(Suppl 2):ii12–24. doi: 10.1093/heapol/czv124 (PMC5009221; doi:10.1093/heapol/czv124)
Supplement: Supplementary Data [file supp_czv124_suppl_data.zip › DistrictDecisionMaking_Paper2_Table4.doc]

**Table 4: Sources of data used for decision-**making

| **Article (ID number, author, date)** | **HMIS data** | **Facility records** | **Document reviews** | **Other sources of data^1^** |
| --- | --- | --- | --- | --- |
| 1. **La Vincente S, et al (2013)** | Yes | Limited | Yes | Special surveys and studies |
| 2. **Mutale W, et al (2013)** | Ghana: Yes | Yes | Yes | No |
|  | Mozambique: No | Yes | No | No |
| 3. **Maluka S, et al (2011a)** 4. **Maluka S, et al (2011b)** 5. **Maluka S, et al (2010)** | Yes | Yes | No | Expert opinion (from workshops), National policy requirements, Conducted survey of priorities/needs of hospitals, health centres, dispensaries and community |
| 6. **Nnaji GA, et al (2008)** | Yes | No | Yes | No |
| 7. **de Savigny D, et al (2008)** | Yes | No | Yes | Demographic Surveillance System |
| 8. **Mutemwa RI, (2006)** | Yes | No | Yes | Observational, Discussion, Experiential (through supervisory visits and consultative visits),  Training |
| 9. **Soeung SC, et al (2006)** | No | No | Yes | Data from CIP micro-plan activities;  Observational description of introduction of a pilot project |
| 10. **Chaulagai CN, et al (2005)** | Yes | Yes | Yes | Findings from an analysis of strengths and weaknesses of the existing information system |
| 11. **Mubyazi G, et al (2004)** | Yes | No | Yes | Studies and information collected by vertical programmes, information through community channels |
| 12. **Heinonen T, et al (2000)** | No | No | No | Conducted household surveys, Focus group discussions, Discussion |
| 13. **Murthy N, (1998)** | Yes | Yes | No | Conducted household and facility surveys, Observation at mother and child protection camps |
| 14. **Sandiford P, et al. (1994)** | Yes | Yes | Yes | Catchment population estimates |

^1^ Brief description of other data sources, where applicable
